# The Doppler Perfusion Index of the Liver and the Underlying Duplex Sonography of Visceral Vessels—A Systematic and Comprehensive Evaluation of Reproducibility

**DOI:** 10.3390/diagnostics14070778

**Published:** 2024-04-08

**Authors:** Christian Lueders, Johannes Gladitz, Georg Bauer, Christian Jenssen, Jana Belaschki, Arndt von Kirchbach, Christoph Schneider, Thomas Kiefer, Heinz Voeller, Daniel Merkel

**Affiliations:** 1 Klinik am See, Rehabilitation Center for Internal Medicine, 15562 Rüdersdorf bei Berlin, Germany; thomas.kiefer-trendelenburg@klinikamsee.com (T.K.); heinz.voeller@klinikamsee.com (H.V.); 2 Brandenburg Medical School (MHB), 16816 Neuruppin, Germany; 3 Statistik-Service Dr. Gladitz, 10119 Berlin, Germany; j.gladitz@t-online.de; 4 Department of General Surgery, Krankenhaus Maerkisch-Oderland, 15344 Strausberg, Germany; g.bauer@khmol.de (G.B.); j.belaschki@khmol.de (J.B.); 5 Department of Internal Medicine, Krankenhaus Maerkisch-Oderland, 15344 Strausberg, Germany; c.jenssen@khmol.de; 6 Brandenburg Institute of Clinical Ultrasound (BIKUS), Medical University Brandenburg, 16816 Neuruppin, Germany; daniel.merkel@immanuelalbertinen.de; 7 Heart Center Brandenburg, Department of Surgery, University Hospital of the Brandenburg Medical School (MHB), 16321 Bernau, Germany; arndt.vonkirchbach@immanuelalbertinen.de; 8 Immanuel Klinik Rüdersdorf, University Hospital of the Brandenburg Medical School (MHB), 15562 Rüdersdorf bei Berlin, Germany; christoph.schneider@immanuelalbertinen.de; 9 Center of Rehabilitation Research, University of Potsdam, 14476 Potsdam, Germany

**Keywords:** Doppler perfusion index (DPI), visceral Doppler, mesenteric Doppler, reproducibility, interobserver variability, Person’s correlation coefficient (PCC), intraclass coefficient (ICC), colorectal cancer, pancreatic ductal adenocarcinoma (PDAC), hepatic metastases

## Abstract

Prior to the curative resection of colorectal carcinoma (CRC) or pancreatic ductal adenocarcinoma (PDAC), the exclusion of hepatic metastasis using cross-sectional imaging is mandatory. The Doppler perfusion index (DPI) of the liver is a promising method for detecting occult liver metastases, but the underlying visceral duplex sonography is critically viewed in terms of its reproducibility. The aim of this study was to investigate systematically the reproducibility of the measured variables, the calculated blood flow, and the DPI. Between February and September 2023, two examinations were performed on 80 subjects within a period of 0–30 days and at two previously defined quality levels, aligned to the German standards of the DEGUM. Correlation analyses were carried out using Pearson’s correlation coefficient (PCC) and the intraclass correlation coefficient (ICC). The diameters, blood flow, and DPI showed a high degree of agreement (PCC of 0.9 and ICC of 0.9 for AHP). Provided that a precise standard of procedure is adhered to, the Doppler examination of AHC, AHP, and PV yields very reproducible blood flows and DPI, which is a prerequisite for a comprehensive investigation of its prognostic value for the prediction of metachronous hepatic metastasis in the context of curatively treated CRC or PDAC.

## 1. Introduction

After the exclusion of metastasis existing at the time of diagnosis of colorectal cancer (CRC) or pancreatic ductal adenocarcinoma (PDAC), resection of the primary tumor is performed. Extensive studies on multimodal management as part of a curative concept have improved the prognosis of these diseases in recent years [1,2,3]. However, a significant proportion of these patients treated with curative intent still suffer a relapse, primarily in the form of hepatic metastasis. This metastasis must have already been present at the time of diagnosis, at a size below the detection limit of sectional imaging, according to the state of the art. Despite the significantly improved spatial resolution of sectional imaging, detection methods for occult liver metastasis, aside from the visualization of a tumor, are still required. A good example of this is the progress made in the field of liquid biopsy [4].

As a further approach in the oncological and visceral surgical context, the Doppler perfusion index (DPI), with its prognostic significance regarding the metachronous manifestation of liver metastases, is a promising instrument. The pathophysiological concept is the very early arterialization of the liver blood flow. It is already shown to be highly significant [5] and has been discussed on a sophisticated level, even up to its predictive value [6]. Technically, the DPI is based on the Doppler sonographic examination of these mesenteric vessels: the common hepatic artery (Arteria hepatica communis, AHC), the proper hepatic artery (Arteria hepatica propria, AHP), and the portal vein (Vena portae, PV). 

Extensive research is devoted to the duplex sonography of mesenteric vessels, and its possible indications aside from the DPI are numerous [7]. Nevertheless, the lack of reproducibility of this admittedly investigator-dependent diagnostic method is often criticized. On the one hand, this is reflected in the cautious clinical use and the status of mesenteric Doppler in current guidelines [8,9]. On the other hand, it is reflected in the fact that all DPI follow-up studies after Leen et al. 1999 demonstrated a trend but were unable to reproduce the prognostic value of the DPI in relation to metachronous liver metastasis with the reported significance [10,11,12,13].

Before systematically investigating the prognostic and possibly predictive value of the DPI, the aim of our study is to systematically investigate the reproducibility of the measured variables, the calculated blood flows, and the DPI. Our aim is also to advance the discourse on the clinical applicability of visceral Doppler.

## 2. Materials and Methods

### 2.1. Operators

All subjects were examined by an experienced examiner with a standard level of training authorized by DEGUM level I (D1) on a standard mid-range ultrasound device (C.L. used a Canon, Aplio i300, Tokyo, Japan). In addition, all subjects were examined in another location by a sonographer with a standard level of training characterized by DEGUM level III (D3) on a high-end ultrasound device (D.M. used a Canon, Aplio i900). For all Doppler studies, a convex probe was used with a range between 1 and 5 MHz.

### 2.2. Subjects

The approval of the local university’s ethics committee and the written consent of the test subjects were obtained. A representative sample of subjects was selected for this study, which corresponds to typical patients in terms of age, disease, treatment concept, and individual concomitant medication who are to be examined in follow-up studies on the clinical value of the DPI. Within 6 months after the curative resection of CRC or PDAC and adjuvant chemotherapy, if necessary, two consecutive visceral Doppler sonographies were performed within a period of 1–30 days. The measurements were preceded by a fasting period of at least 8 h. All individual long-term medications (including antihypertensives and frequency-limiting medications) were taken as usual. The examinations were carried out with patients in the supine position and under respiratory suspension during expiration.

### 2.3. Investigation Procedure

#### 2.3.1. Determination of Vessel Diameter

**Figure 1:**
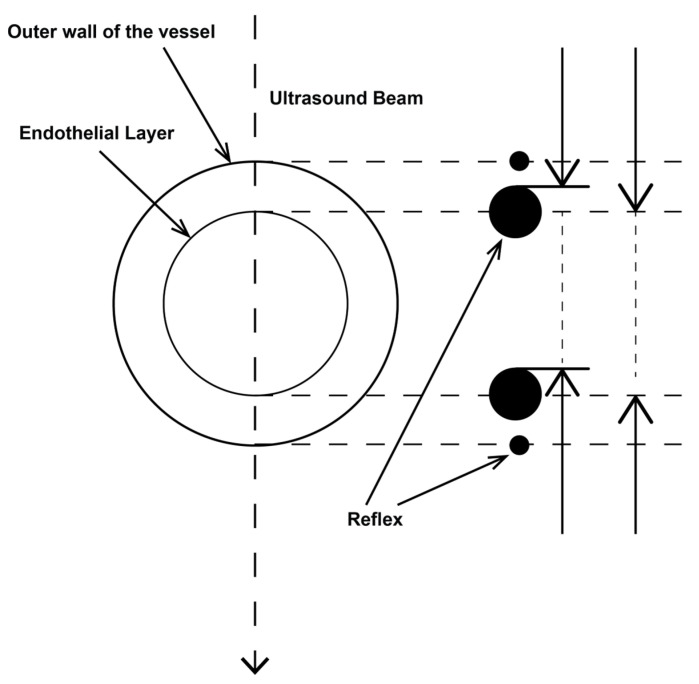
Leading edge method.

Determining the vessel diameter to calculate blood flow is a critical issue, especially for very small vessels such as the AHP. Inaccuracies are squared when calculating the cross-section. The AHC should be measured after leaving the truncus, when the vessel course is straight for the first time. The diameter of the AHP can be measured either from the upper abdominal cross-section or from the intercostal view. Since the Doppler profile from the intercostal view is favorable, but the vessel diameter sometimes cannot be effectively captured in this location, the vessel diameter can also be captured in the upper abdominal cross-section, just before the bifurcation to the right and left lobes of the liver. The portal vein was always recorded from an intercostal area, and the vessel diameter was reliably determined at the level of entry into the liver parenchyma and always before bifurcation. **Blooming effect**. The exact determination of the inner diameter of small vessels is complicated by the reflection of ultrasound at the interfaces of tissues with different physical properties (e.g., vessel wall and blood, intima, and media). This so-called “blooming effect” leads to strong echoes, which exceed the anatomical size and, thus, make the vessel walls appear thicker than they really are [14]. The background is an incident sound wave that is partially reflected at an interface and can be partially or fully reflected or transmitted into the neighboring layer. This error can be countered by measuring from the outer to the inner reflex of the inner vessel wall (Figure 1).

For larger vessels with a diameter of at least 10 mm, such as the portal vein, measuring the internal distance of the internal reflections leads to more accurate values. In the present study, the leading edge method was consistently applied to the arterial vessels by using sectional enlargement. Sectional enlargement was also used for the portal veins. **Time dependence**. An incorrect determination of the vessel diameter can also occur if the vessel diameter is not constant over time. The diameter of the portal vein depends on respiration, and, therefore, the diameter of the PV was measured under respiratory suspension during expiration. Despite numerous methods described in the literature for determining a mean vessel diameter to account for caliber fluctuations over time, the authors of this study did not see any relevant pulsatility in either AHC or AHP [15,16].

#### 2.3.2. Location of the Probe, Insonation Angle, and Doppler Window

**Figure 2:**
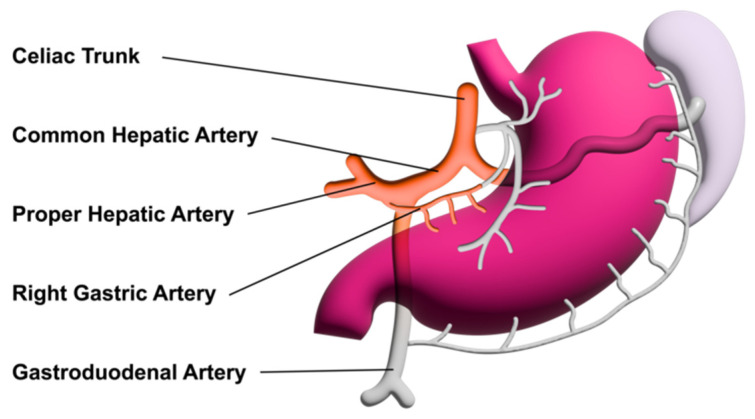
Arterial blood supply to the liver.

Figure 2 shows the normal anatomy of the arterial liver supply.

**Figure 3:**
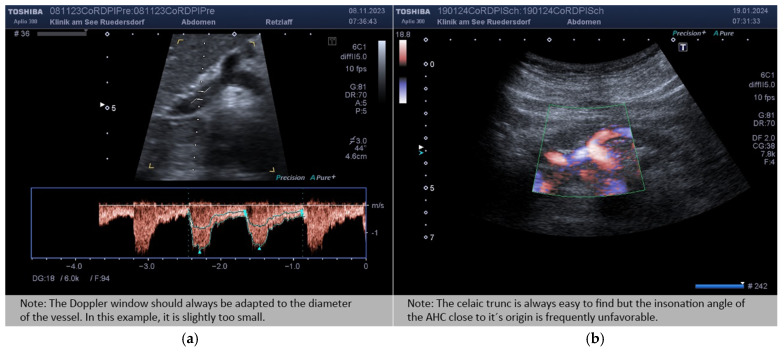
Insonation of AHC close to its origin. (**a**) Good insonation angle. (**b**) Angle is too flat (greater than 60°).

The insonation angle refers to the angle between the vessel to be measured and the transducer. **Common Hepatic Artery:** The outflow of the AHC from the celiac trunk was identified in an upper abdominal cross-section. In this context, vascular variants of the arterial liver supply were sought, particularly with regard to an additional supply to the left lobe of the liver. In the case of an additional vessel, this circumstance was documented (about 5% of cases). However, the subjects were not excluded from the study. The Doppler gate was adjusted to the diameter of the vessel so that it covered about three-quarters of the lumen. It was positioned at least 1 cm from the origin of the AHC in a straight section of at least 2 cm and with as acute an angle as possible. The literature describes positioning the Doppler gate as close as possible to the origin. In our investigations, this position led to clearly different spectral patterns in the sense of the flow profile of the celiac trunk [12,17]. These ideal conditions could not always be fulfilled (Figure 3).

Nevertheless, Doppler measurements that were recorded at an angle of more than 60° were not considered further (but the diameter of the vessel was). Each measurement was repeatedly performed until a spectral pattern of good quality was obtained at least three times.

**Figure 4:**
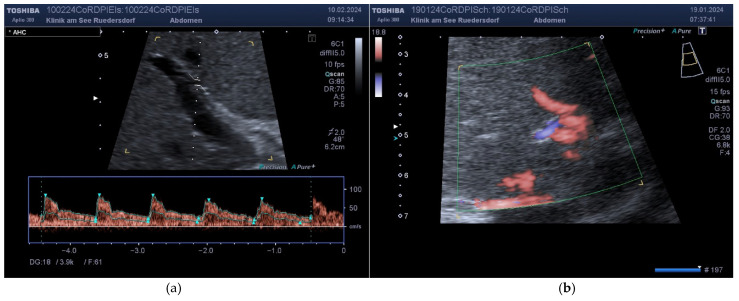
Example of an AHP with a straight course. (**a**) Doppler with the use of section enlargement. (**b**) Identification of bifurcation.

**Proper Hepatic Artery:** The AHP was then followed to identify the origin of the gastroduodenal artery and the bifurcation of the AHP. It was possible to find the origin of the right gastric artery in a significant proportion of the test subjects. Within the subjects, after resection of the pancreatic head (Whipple operation, PPPD), it was assumed that at least the gastroduodenal artery could no longer be identified. In the majority of cases, the diameter of the AHP can be reliably determined prior to bifurcation (Figure 4). The Doppler measurement of the AHP itself was primarily performed from an intercostal view. Here, it was also necessary to obtain several measurements with a good spectral pattern on a straight section of at least 1 cm. This anatomy allowed for much better angles (Figure 4).

**Figure 5:**
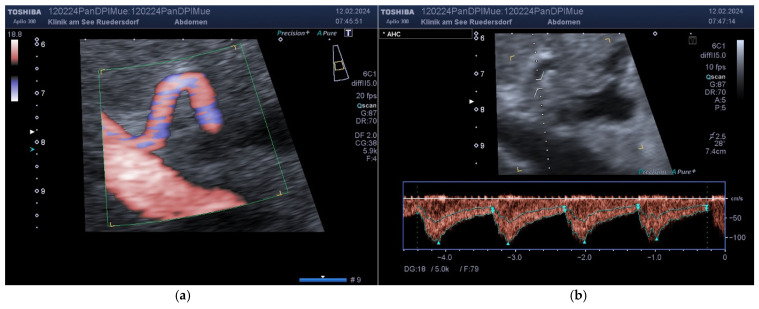

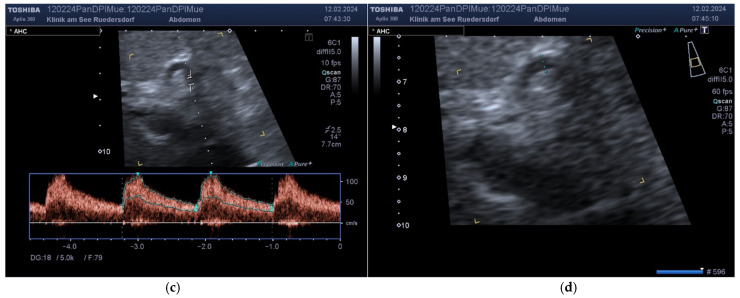
Example of an AHP with a meandering course. (**a**) Identification with section enlargement. (**b**) Doppler. (**c**) Additional Doppler in a further course. (**d**) Determination of diameter (leading edge method).

Nevertheless, not all quality criteria could always be met with this vessel either (Figure 5).

**Figure 6:**
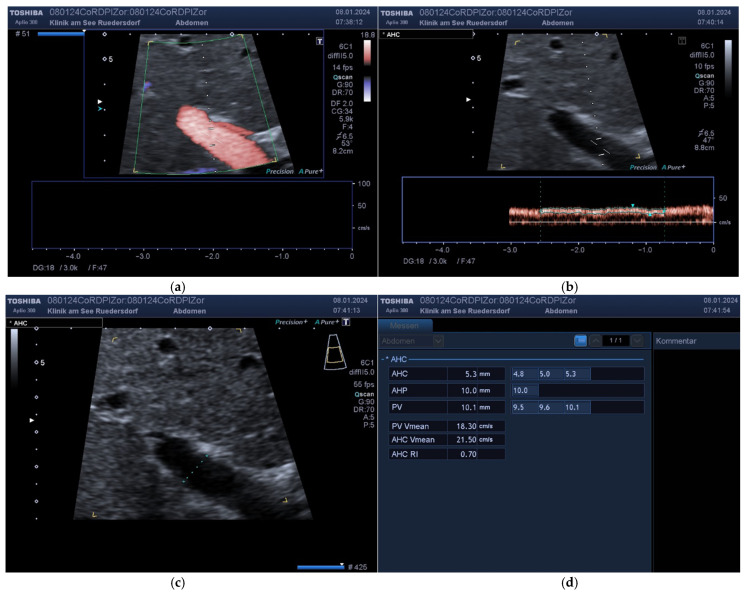
Measurement of a PV. (**a**) Color. (**b**) Doppler with a window corresponding to the diameter. (**c**) Determination of the diameter. (**d**) Documentation of the results in the preset.

**Portal Vein:** Measuring the blood flow of the PV is part of the internist’s daily clinical routine and is rarely a challenge. Without exception, these measurements were also taken from the intercostal view (Figure 6).

### 2.4. Variables Acquired and Calculation of Blood Flows and DPI

After the exclusion of a currently present hepatic metastasis in the B-Mode, the following parameters were measured using Doppler sonography:

- The vessel diameter (*d*, mm);
- The insonation angle (*θ*, degrees);
- The Doppler shift (*V*, cm/s);
- The resistive index for AHC and AHP (RI, non-dimensional).

The vessel diameter of the respective vessel was determined at least three times in each examination, and the arithmetic mean was calculated. Duplex sonography of the respective vessel was also performed at least three times. The measurements with the most acute insonation angle were evaluated. An equally important criterion was the quality of the spectral pattern. The arithmetic mean was then determined for measurements of equal value under these criteria.

The blood flow (mL/min) in the respective vessel was then calculated within the database using the following formula:

$$F=\frac{V\cdot \frac{\pi }{4}\cdot d^{2}\cdot 60}{100\cdot \cos\left(\theta \right)}$$

Finally, the *DPI* was calculated as a function of the respective arterial vessel as follows:

$$DPI\;(HA)=\frac{F(HA)}{F\left(HA\right)+F(PV)}$$

*F*: blood flow; *HA*: hepatic artery (AHC or AHP); *PV*: portal vein.

The blood flow through the respective arterial vessel was set in relation to the sum of the blood flow through the arterial vessel and the blood flow through the portal vein. This results in a dimensionless quotient with values greater than zero and a maximum of one.

### 2.5. Statistics

The sample size was empirically determined. We found one publication that focused on the reproducibility of the DPI, with a study design that is still the most comparable. This study had a sample size of *n* = 20 and is already over 20 years old. A sample size twice as large (*n* = 40) was empirically determined following approval from the local ethics committee. This should apply to both disease entities (CRC, PDAC), as local experts suspected that subjects after surgery in the pancreatic head area may have had a significantly more difficult sonographic situs. The following steps were taken for statistical analysis:

(a) Test for normal distribution;
(b) Test for differences in means between the two operators (systematic bias) using the exact Wilcoxon test for paired samples;
(c) Test for equality of variances between the two observers (Levene test) (if there is high correlation, mean, and variance equivalence, there is a high absolute agreement);
(d) Calculation of Pearson’s correlation coefficient between the measurements of both operators;
(e) Calculation of the intraclass correlation coefficients under the assumption that the absolute agreement is tested; the raters were selected as two raters from the population of all potential raters, and the patients are random (two-way random model). The ICC for single measurement is reported;
(f) Calculation of the mean coefficient of variation (MVC) across all patients (the standard deviation of both measurements per patient divided by the mean of both measurements).

The Bland–Altman plot and the PCC were used to measure the agreement between the paired measured and the calculated values. The PCC is a particularly suitable method for displaying the agreement of paired measured values. However, a consistent interobserver bias may not be detected. Therefore, a significant systematic interobserver bias was excluded by using the exact Wilcoxon test before the analysis.

Outliers can occasionally be seen in the scatterplots. These can be caused by measurement errors, input errors, or unusual fluctuations in the values for individual patients. These were checked and, where possible, corrected. The elimination of outliers from the data was deliberately omitted because they occur in clinical practice.

## 3. Results

### 3.1. Descriptive Data and Indices

A total of *n* = 80 patients were included, and they were measured and evaluated by both investigators. The sample included 40 patients after CRC (14 men, 26 women, mean age 63.7 ± 11.7 years) and 40 patients after PDAC (21 men, 19 women, mean age 69.1 ± 8.9 years). Of all patients, 56.3% were female and 43.8% were male (*p* = 0.176). Patients in the pancreatic cancer group (PDAC) were significantly older with *p* = 0.017 (63.7 ± 11.7 years vs. 69.1 ± 8.9 years), while BMI was significantly higher in patients with colon cancer (26.1 ± 3.9 kg/m^2^ vs. 23.0 ± 4.7 kg/m^2^ with *p* = 0.001). There were no significant differences in total protein, while albumin was significantly higher in the CRC group (42.3 ± 5.0 g/L vs. 39.1 ± 3.9 with *p* = 0.001). For sonographic Doppler measurements, the insonation angle used is a known source of error. This angle should be as small as possible and not exceed a value of 60°. All measurements for which an angle greater than 60° was used were discarded. Consequently, not all 80 data pairs could always be used, and in such cases, the number of cases was lower.

### 3.2. Measured Values and Calculated Blood Flow

Descriptive data and indices for PV, AHC and AHP are presented in Table 1, Table 2 and Table 3. Bland–Altman plots of the paired values for vessel diameter, insonation angle, Doppler shift, blood flow, and DPI of all three vessels are provided in the Supplementary Materials (Figures S1–S16).

Scatterplots of the measured values were used as graphical methods, comparing the measurements of Operator 1 (DEGUM 1) and Operator 2 (DEGUM 3), with a 45° line drawn as the line of absolute agreement. The standard deviation between two measurements on the same patient is exactly the orthogonal distance of the two-dimensional measurement point from the 45° line in the scatter diagram. This distance is then divided by the mean value of both measurements, thus achieving scale independence. The mean value of all the patient-related coefficients of variation is reported.

**Figure 7:**
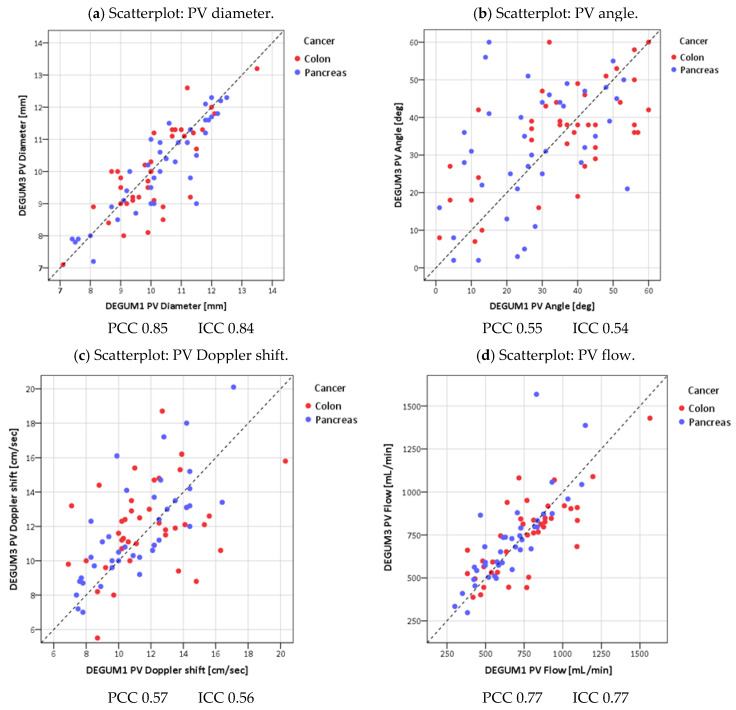
Portal vein.

**Figure 8:**
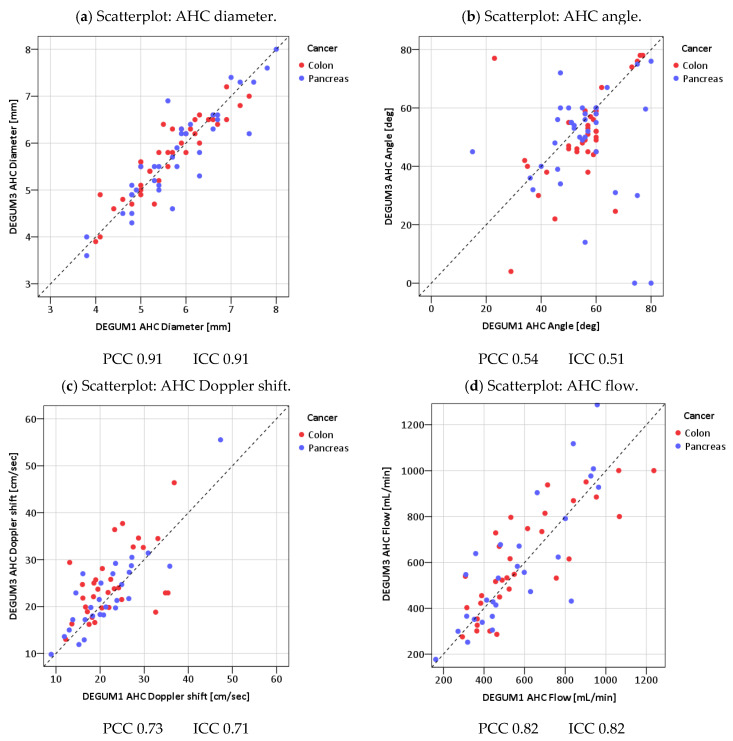
Common hepatic artery.

**Figure 9:**
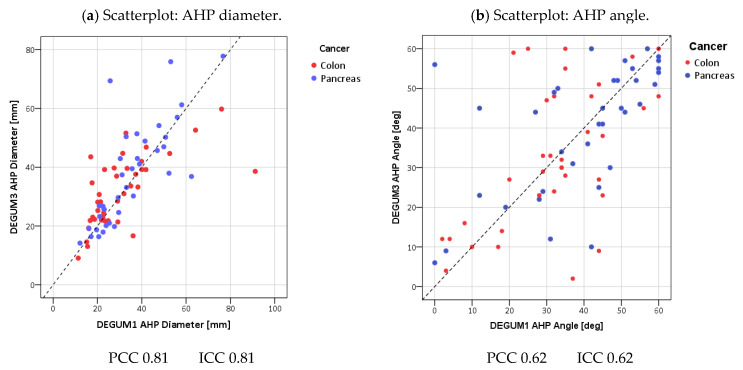

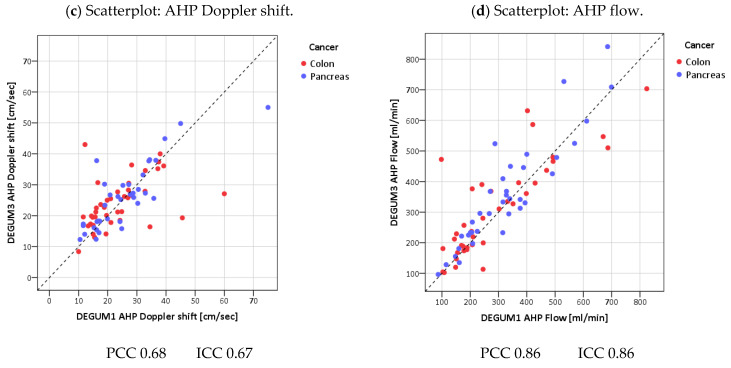
Proper hepatic artery.

The scatterplots of the vessel diameters of all three vessels show very good agreement (vessel diameter PV: Pearson 0.85; ICC 0.84; AHC Pearson 0.91; ICC 0.91; AHP Pearson 0.81; ICC 0.81) (Figure 7, Figure 8 and Figure 9). The direct comparison of the paired values for insonation angle and Doppler shift shows only moderate agreement (insonation angle: PV Pearson 0.55; ICC 0.54; AHC Pearson 0.54; ICC 0.51; AHP Pearson 0.62; ICC 0.62; Doppler shift: PV Pearson 0.57; ICC 0.56; AHC Pearson 0.73; ICC 0.71; AHP Pearson 0.68; ICC 0.67) (Figure 7, Figure 8 and Figure 9). This shows the subjectivity of the examination. However, it would be a mistake to assume that this would also result in greater uncertainty of the calculated values. In fact, insonation angle and Doppler shift in pairs lead to repeatedly equal blood flows (blood flow PV Pearson 0.77; ICC 0.77; AHC Pearson 0.82; ICC 0.82; AHP Pearson 0.87; ICC 0.86) (Figure 7, Figure 8 and Figure 9).

### 3.3. DPI

**Figure 10:**
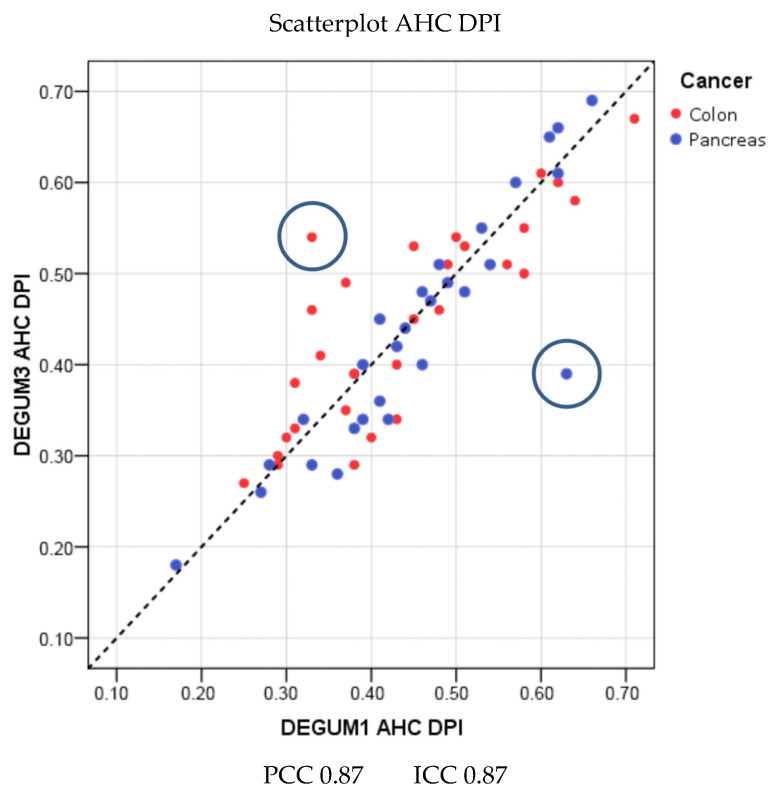
Common hepatic artery.

**Figure 11:**
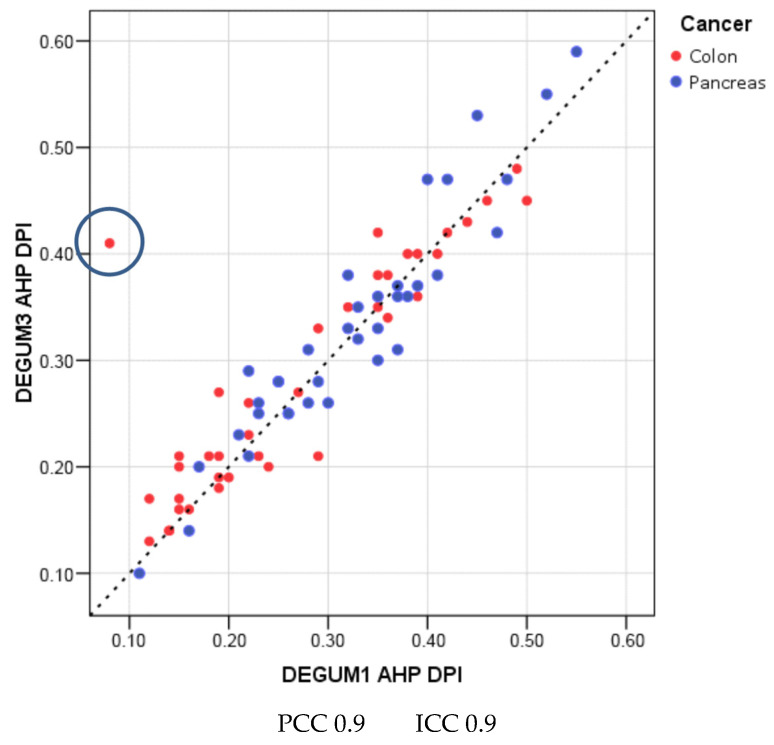
Proper hepatic artery.

The scatterplots of the DPIs for AHC and AHP calculated from the blood flows show very good reproducibility (DPI AHC: Pearson 0.87 ICC 0.87; AHP: Pearson 0.9 ICC 0.9) (Figure 10 and Figure 11).

**Figure 12:**
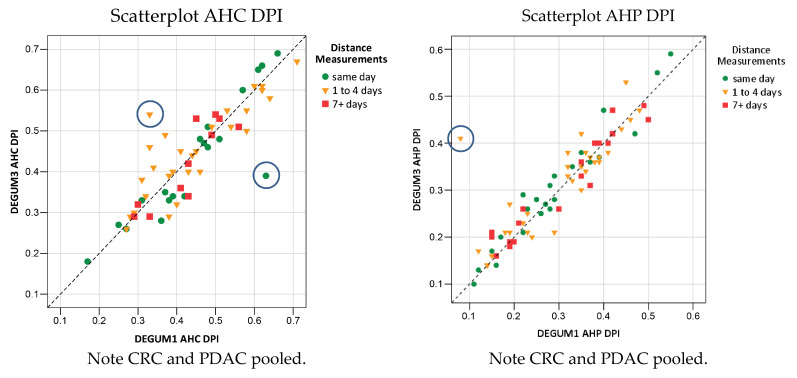
DPI and the question of time dependency.

As described in the methods section, outliers were left in calculations and illustrations (Figure 10, Figure 11 and Figure 12). The study is intended to reflect everyday clinical practice rather than generate hypotheses.

### 3.4. Resistive Index

**Figure 13:**
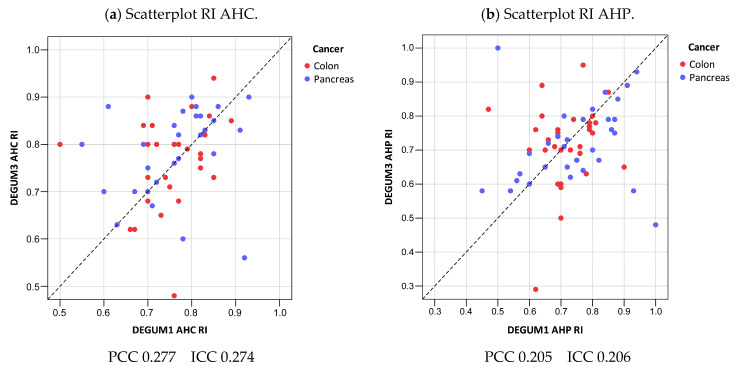
Resistive index.

Since the determination of the vessel diameter and the Doppler examination of the AHP are considerably more difficult, the resistive index (RI) is often mentioned in the literature as a more reliable surrogate parameter. Our study cannot confirm this (resistive index: AHC Pearson 0.277 ICC n.d.; AHP Pearson 0.205 ICC n.d.) (Figure 13).

### 3.5. The Question of Time Dependency

Most of the test subjects were not seen by the two investigators on the same day. The two measurements (DEGUM 1 and DEGUM 3) were, therefore, carried out from 0 to 30 days apart. In addition, the DEGUM 1 measurements were mainly performed early in the morning and the DEGUM 3 measurements mostly at midday. This raised the question of whether the time of day, on the one hand, and the time interval in particular, had an influence on the reproducibility of the measurement data. The measurement data were categorized into the following groups: measurement on the same day (Figure 12, green), measurement at an interval of 1 to 4 days (Figure 12, orange), and measurement at an interval of 7 or more days (Figure 12, red). No subjects were measured with a time interval of 5 or 6 days.

The categorical, colored resolution graphically shows that, under the test conditions described above, the time of day and a measurement interval of up to 30 days have no relevant influence on the DPI for AHC or AHP. A separate statistical analysis in relation to the time intervals was not carried out, and only a differentiation by color was performed in this study.

## 4. Discussion

In the context of the dual blood supply to the liver, the blood flow through the hepatic artery is set in relation to the total blood flow, i.e., the sum of arterial and portal venous inflow. It is postulated that, in certain liver diseases, in the presence of liver metastases, the blood flow shifts towards the arterial part. In the case of a significant proportion of liver metastases in the total mass of the liver, this shift has already been demonstrated, and its pathophysiology was explained in detail as early as the 1950s [18]. The described shift in very early metastasis, which is not yet detectable using imaging (i.e., it is occult), cannot be explained by the mass effect alone but has also been described in detail [6].

In the curative setting of CRC or PDAC, despite high-resolution cross-sectional imaging at the time of resection, there is uncertainty as to whether occult (liver) metastasis already exists. Using the Doppler perfusion index of the liver, early detection should be possible due to the arterialization of the liver perfusion already existing at the stage of microscopic metastasis. An often-criticized weakness of this examination method is the frequently described subjectivity of the underlying visceral Doppler. This is reflected in the discrepancy between the many possible applications of the visceral Doppler and its actual significance in clinical use and in the guidelines (Guidelines for CRC, PDAC, and cirrhosis). The pitfalls of this investigation are described in detail in the literature. Studies on reproducibility and attempts to standardize this method have already been undertaken [19]. Some of these studies date from an era that does not correspond to the current state of the art (both in the fields of cross-sectional imaging and Doppler sonography), and they consistently lack sufficient scope and stringent analysis. Consequently, further studies were recommended to address these problems.

A correct step in constructively dealing with the Achilles’ heel of visceral Doppler is described in Ignee et al. 2016 [7]. Here, the factors that can be influenced are differentiated from those that cannot. The fact that in previous studies more than 20% of the measurements could not be evaluated due to poor quality [13] emphasizes the most important influenceable factor: the expertise of the examiner.

A study on the reproducibility of the DPI and the parallel evaluation of the underlying measured values of visceral Doppler, especially in a representative sample of sufficient size, has not been published thus far. The current study is an important step toward a comprehensive investigation of the value of DPI in the window of uncertainty after curative therapy for CRC or PDAC.

By following a strict protocol with respect to diameter and vessel type (artery or vein) in our study, the reproducibility of vessel diameters was excellent, especially with an emphasis on the leading edge method. The analysis of the agreement between the angle of insonation and the Doppler profile dependent on it shows the subjectivity of this examination. Here, the analysis showed a moderate agreement at best. Even if the anatomy of the individual remains unchanged, the experienced examiner adjusts the probe in such a way that they can perform the most accurate measurement. This means that angles close to or above 60° should be avoided. In this study, measurements at an angle of over 60° were not evaluated. The primary goal of each measurement was to obtain a good spectral pattern (at as acute an angle as possible) in a controlled manner. Our measurements were time-consuming and sometimes took up to 45 min per session. Under these conditions, particularly after pancreatic head resection, intestinal gas cannot be accepted as an obstacle to the visualization of AHC or AHP. If the calculation of the blood flows (and, thus, the DPIs) is based on high-quality measurements, as in the case of our study, this is reflected in the excellent reproducibility achieved, with a PCC or an ICC of 0.9 or more.

Regarding the correlation analyses, the intraclass correlation, or the intraclass correlation coefficient (ICC), is a frequently used method for testing the correlation of values [20,21]. Although it operates on data structured as groups rather than as paired observations, this method has been applied in the context of high-quality studies on similar issues [22]. Pearson’s correlation is described in the literature as less suitable for correlation analyses such as ours [22]. The authors consider it to be quite suitable, provided that there is no bias between the operators and the variances do not differ. Due to the variable use of these correlation analyses, our calculations were carried out using both Pearson correlation and ICC. Both analyses led to highly consistent values. Thus, after many discussions with our statistician and extensive research of the relevant literature, we can be assured that both methods can be used for questions such as ours.

## 5. Conclusions

Our study shows that the visceral Doppler for calculating blood flow in AHC, AHP, and PV can be a reliable and reproducible test to conclusively re-examine the prognostic value of DPI in relation to the development of liver metastases.

In the visceral Doppler of the vessels treated here, obtaining measurement data of good and, thus, reproducible quality is less dependent on the availability of a high-end device. However, years of experience in ultrasound and the willingness to implement the rules known in vascular diagnostics in a disciplined manner are indispensable.

## Figures and Tables

**Table 1:** Vena portae (PV): descriptive data and indices.

Parameter	Diameter [mm]	Insonation Angle	Doppler Shift [cm/s]	Flow [mL/min]
N	79	79	79	79
Mean D1 (SD)	10.3 (1.3)	31.7 (16.2)	11.5 (2.7)	712 (236)
Mean D3 (SD)	10.1 (1.4)	34.0 (14.9)	11.9 (2.3)	729 (238)
Mean tot (SD)	10.2 (1.3)	32.8 (15.5)	11.7 (2.7)	720 (237)
Mean Diff (SD)	−0.15 (0.74)	2.4 (14.8)	0.4 (2.5)	17 (160)
p(Bias)	0.20	0.26	0.11	0.55
p(Var)	0.57	0.29	0.81	0.82
PCC	0.85	0.55	0.57	0.77
ICC (95% CI)	0.84 (0.77; 0.90)	0.54 (0.37; 0.68)	0.56 (0.39; 0.70)	0.77 (0.67; 0.85)
MVC	0.04	0.33	0.12	0.11
B/A Limits	(−1.6; 1.3)	(−26.7; 31.4)	(−4.5; 5.3)	(−297; 331)

Note: N = sample count; D1 = rater DEGUM 1; D3 = rater DEGUM 3; p(Bias) = significance of a bias (Wilcoxon), test for equality of means using the exact non-parametric Wilcoxon test; p(Var) = significance of the test for equality of variances between raters; r = Pearson’s correlation; ICC = intraclass correlation; MVC = mean variation coefficient; B/A Limits = Bland–Altman limits; Mean tot = mean of D1 + D3; SD = standard deviation.

**Table 2:** Arteria hepatica communis (AHC): descriptive data and indices.

Parameter	Diameter [mm]	Insonation Angle	Doppler Shift [cm/s]	Flow [mL/min]	AHC-DPI
N	75	60	60	60	60
Mean D1 (SD)	5.7 (1)	52.1 (9.2)	22.1 (7.3)	579 (240)	0.4 (0.1)
Mean D3 (SD)	5.8 (0.9)	48.4 (11.4)	23.8 (8.1)	598 (252)	0.4 (0.1)
Mean tot (SD)	5.7 (0.9)	50.2 (10.5)	23 (7.7)	588 (245)	0.4 (0.1)
Mean Diff (SD)	0.0 (0.4)	−3.7 (10)	1.7 (5.7)	20 (148)	0.01 (0.06)
p(Bias)	0.50	0.002	0.008	0.30	0.55
p(Var)	0.97	0.29	0.73	0.70	0.82
PCC	0.91	0.54	0.73	0.82	0.87
ICC (95% CI)	0.91 (0.86; 0.94)	0.51 (0.24; 0.67)	0.71 (0.55; 0.82)	0.82 (0.72; 0.89)	0.87 (0.79; 0.92)
MVC	0.04	0.13	0.13	0.14	0.07
B/A Limits	(−0.78; 0.82)	(−23.3; 16.0)	(−9.4; 12.9)	(−270; 310)	(−0.13; 0.12)

Note: N = sample count; D1 = rater DEGUM 1; D3 = rater DEGUM 3; p(Bias) = significance of a bias (Wilcoxon), test for equality of means using the exact non-parametric Wilcoxon test; p(Var) = significance of the test for equality of variances between raters; r = Pearson correlation; ICC = intraclass correlation; MVC = mean variation coefficient; B/A Limits = Bland–Altman limits; Mean tot = mean of D1 + D3; SD = standard deviation.

**Table 3:** Arteria hepatica propria (AHP): descriptive data and indices.

Parameter	Diameter [mm]	Insonation Angle	Doppler Shift [cm/s]	Flow [mL/min]	AHP-DPI
N	79	76	76	76	76
Mean D1 (SD)	4.4 (0.7)	35.8 (16.9)	24.6 (11.3)	308 (165)	0.3 (0.1)
Mean D3 (SD)	4.5 (0.7)	36.6 (17.3)	25 (9.4)	332 (166)	0.3 (0.1)
Mean tot (SD)	4.5 (0.7)	36.2 (17)	24.8 (10.3)	320 (165)	0.3 (0.1)
Mean Diff (SD)	0.1 (0.4)	0.8 (14.9)	0.5 (8.5)	24 (86)	0.01 (0.05)
p(Bias)	0.10	0.98	0.14	0.05	0.06
p(Var)	0.38	0.33	0.25	0.94	0.66
PCC	0.81	0.62	0.68	0.87	0.90
ICC (95% CI)	0.81 (0.71; 0.87)	0.62 (0.46; 0.74)	0.67 (0.52; 0.78)	0.86 (0.78; 0.91)	0.90 (0.84; 0.93)
MVC	0.05	0.27	0.15	0.13	0.08
B/A Limits	(−0.79; 0.94)	(−28.5; 30.1)	(−16.1; 17.0)	(−145; 192)	(−0.09; 0.11)

Note: N = sample count; D1 = rater DEGUM 1; D3 = rater DEGUM 3; p(Bias) = significance of a bias (Wilcoxon), test for equality of means using the exact non-parametric Wilcoxon test; p(Var) = significance of the test for equality of variances between raters; r = Pearson correlation; ICC = intraclass correlation; MVC = mean variation coefficient; B/A Limits = Bland–Altman limits; Mean tot = mean of D1 + D3; SD = standard deviation.

## Data Availability

The data presented in this study are available on request from the corresponding author. The data are not publicly available due to institutional and national data policy restrictions imposed by the ethics committee, since the data contain information that could potentially identify study participants. Data are available upon request (contact via christian.lueders@klinikamsee.com) for researchers who meet the criteria for access to confidential data (please provide the manuscript title with your inquiry).

## Supplementary Materials

The following supporting information can be downloaded at: https://www.mdpi.com/article/10.3390/diagnostics14070778/s1, Figure S1: PV Bland-Altman plot for diameter; Figure S2: PV Bland-Altman plot for insonation angle; Figure S3: PV Bland-Altman plot for Doppler shift; Figure S4: PV Bland-Altman plot for blood flow; Figure S5: AHC Bland-Altman plot for diameter; Figure S6: AHC Bland-Altman plot for insonation angle; Figure S7: AHC Bland-Altman plot for Doppler shift; Figure S8: AHC Bland-Altman plot for blood flow; Figure S9: AHC Bland-Altman plot for Resistive Index; Figure S10: AHC Bland-Altman plot for DPI; Figure S11: AHP Bland-Altman plot for diameter; Figure S12: AHP Bland-Altman plot for insonation angle; Figure S13: AHP Bland-Altman plot for Doppler shift; Figure S14: AHP Bland-Altman plot for blood flow; Figure S15: AHP Bland-Altman plot for Resistive Index; Figure S16: AHP Bland-Altman plot for DPI.
