# Supplementary material for: The Doppler Perfusion Index of the Liver and the Underlying Duplex Sonography of Visceral Vessels—A Systematic and Comprehensive Evaluation of Reproducibility
Source: Diagnostics (Basel). 2024 Apr 8;14(7):778. doi: 10.3390/diagnostics14070778 (PMC11012103; doi:10.3390/diagnostics14070778)
Supplement: Supplementary file 1 [file diagnostics-14-00778-s001.zip › diagnostics-2924336-supplementary.pdf]

Figure S1

PV Bland-Altman plot for diameter

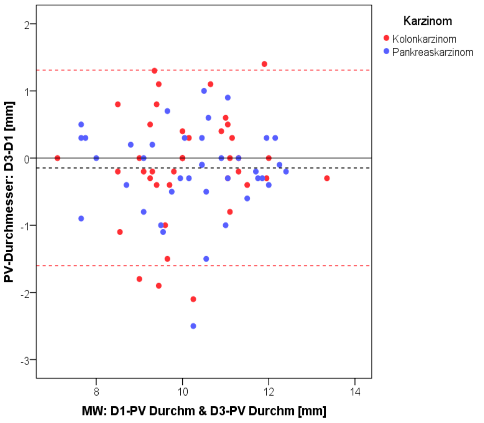

Note. Labeling in German: Kolonkarzinom=CRC, Pankreaskarzinom=PDAC, Durchmesser=diameter

Figure S2

PV Bland-Altman plot for insonation angle

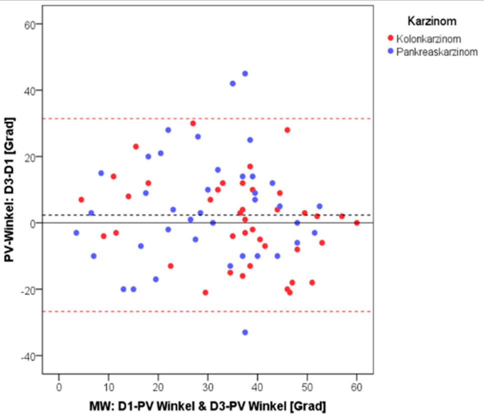

Note. Labeling in German: Kolonkarzinom=CRC, Pankreaskarzinom=PDAC, Winkel=insonation angle

Figure S3

PV Bland-Altman plot for Doppler shift

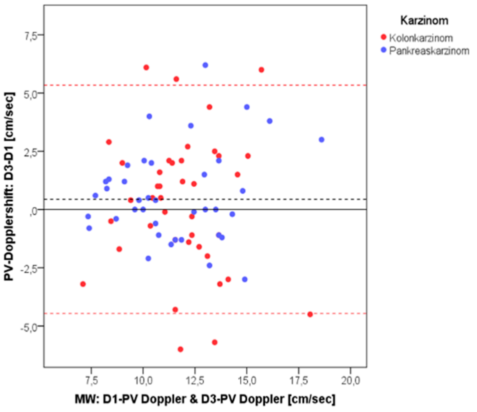

Note. Labeling in German: Kolonkarzinom=CRC, Pankreaskarzinom=PDAC, Dopplershift=Doppler shift

Figure S4

PV Bland-Altman plot for blood flow

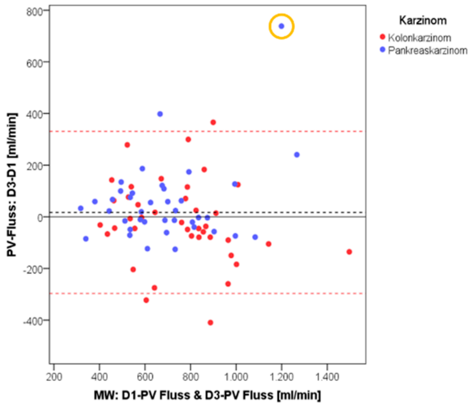

Note. Labeling in German: Kolonkarzinom=CRC, Pankreaskarzinom=PDAC, Fluss=blood flow

Figure S5

AHC Bland-Altman plot for diameter

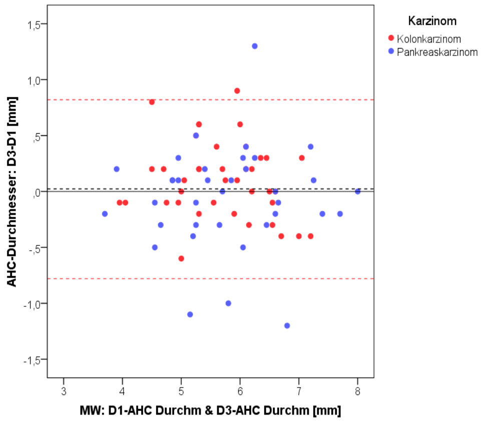

Note. Labeling in German: Kolonkarzinom=CRC, Pankreaskarzinom=PDAC, Durchmesser=diameter

Figure S6

AHC Bland-Altman plot for insonation angle

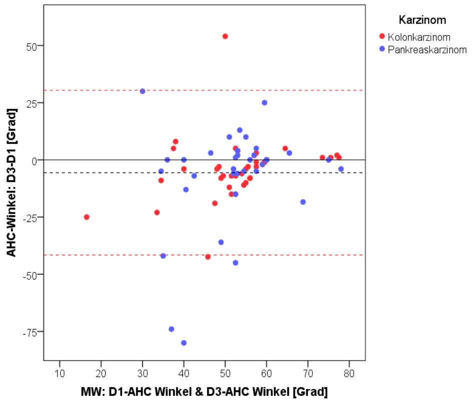

Note. Labeling in German: Kolonkarzinom=CRC, Pankreaskarzinom=PDAC, Winkel=insonation angle

Figure S7

AHC Bland-Altman plot for Doppler shift

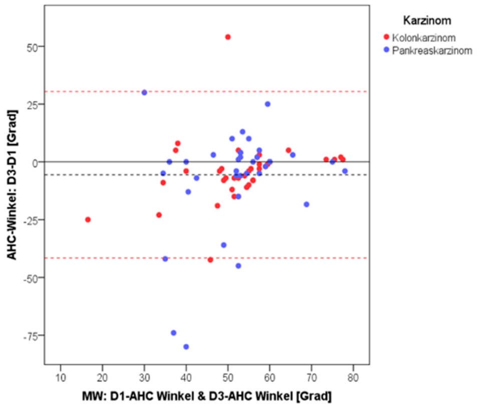

Note. Labeling in German: Kolonkarzinom=CRC, Pankreaskarzinom=PDAC, Dopplershift=Doppler shift

Figure S8

AHC Bland-Altman plot for blood flow

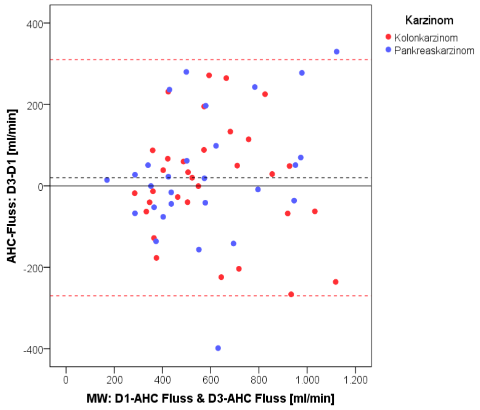

Note. Labeling in German: Kolonkarzinom=CRC, Pankreaskarzinom=PDAC, Fluss=blood flow

Figure S9

AHC Bland-Altman plot for Resistive Index

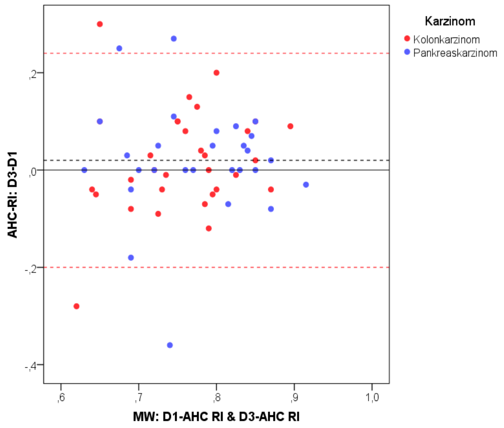

Note. Labeling in German: Kolonkarzinom=CRC, Pankreaskarzinom=PDAC

Figure S10

AHC Bland-Altman plot for DPI

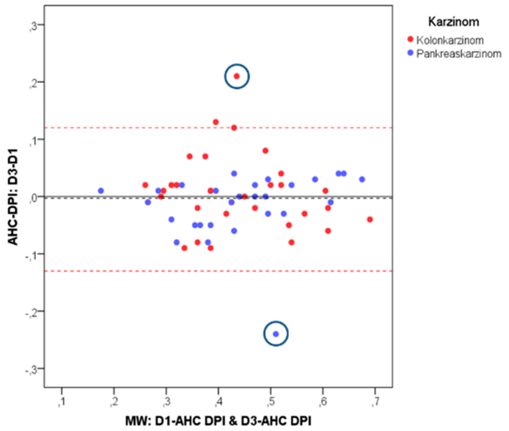

Note. Labeling in German: Kolonkarzinom=CRC, Pankreaskarzinom=PDAC

**Figure S11**
AHP Bland-Altman plot for diameter

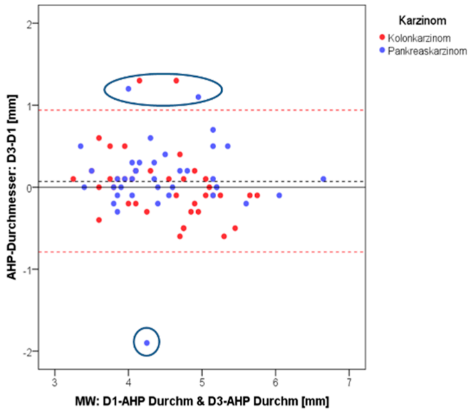

Note. Labeling in German: Kolonkarzinom=CRC, Pankreaskarzinom=PDAC, diamteer=Durchmesser

**Figure S12**
AHP Bland-Altman plot for insonation angle

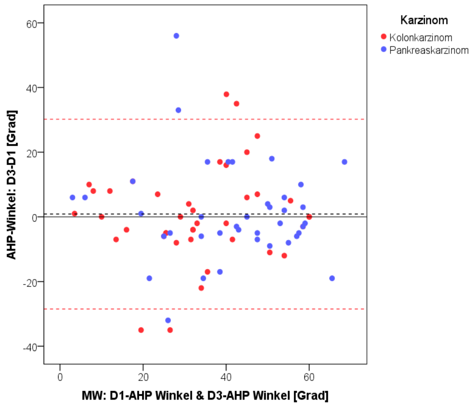

**Figure S13**
AHP Bland-Altman plot for Doppler shift

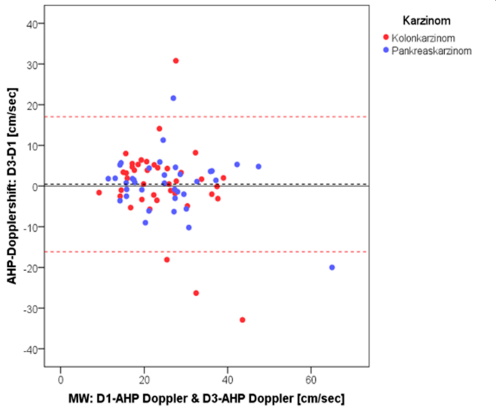

Note. Labeling in German: Kolonkarzinom=CRC, Pankreaskarzinom=PDAC, Dopplershift= Doppler shift

Figure S14

AHP Bland-Altman plot for blood flow

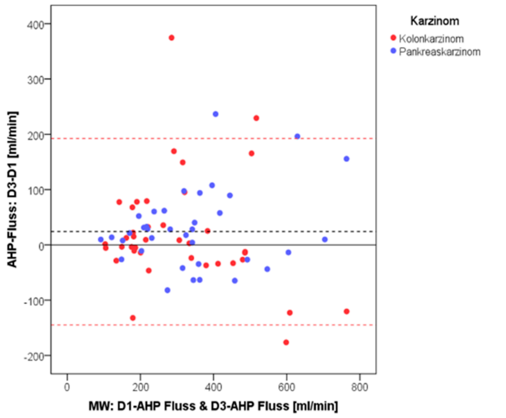

Note. Labeling in German: Kolonkarzinom=CRC, Pankreaskarzinom=PDAC, Fluss= blood flow

Figure S15

AHP Bland-Altman plot for Resistive Index

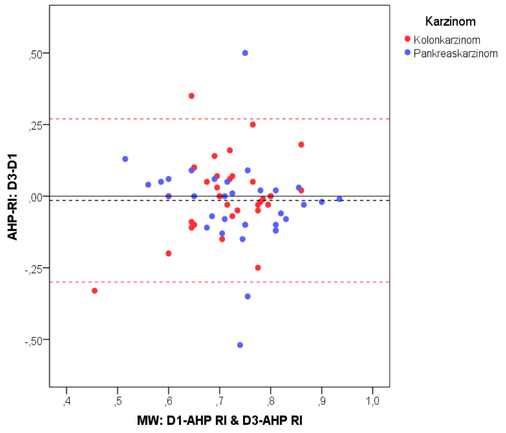

Note. Labeling in German: Kolonkarzinom=CRC, Pankreaskarzinom=PDAC

Figure S16

AHP Bland-Altman plot for DPI

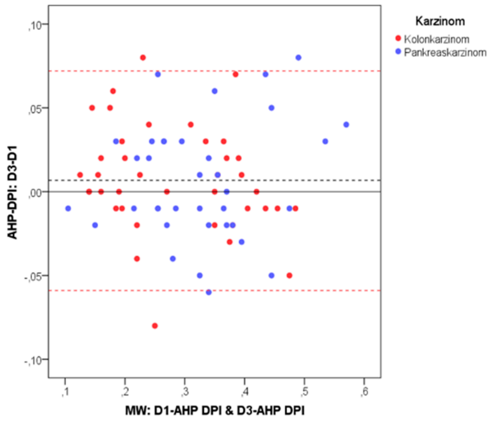

Note. Labeling in German: Kolonkarzinom=CRC, Pankreaskarzinom=PDAC
